# The Quality of Tuberculosis Care in Urban Migrant Clinics in China

**DOI:** 10.3390/ijerph15092037

**Published:** 2018-09-18

**Authors:** Hao Xue, Jennifer Hager, Qi An, Kai Liu, Jing Zhang, Emma Auden, Bingyan Yang, Jie Yang, Hongyan Liu, Jingchun Nie, Aiqin Wang, Chengchao Zhou, Yaojiang Shi, Sean Sylvia

**Affiliations:** 1 School of Economics and Management, Northwest University, Xi’an 710069, China; xuehjjx@gmail.com; 2 Department of Health, Sport, and Exercise Sciences, School of Education, University of Kansas, Lawrence, KS 66045, USA; j829h282@ku.edu; 3 Center for Experimental Economics in Education, Shaanxi Normal University, Xi’an 710127, China; anqiceee@163.com (Q.A.); liukaiceee@163.com (K.L.); eauden@126.com (E.A.); jyang0716@163.com (J.Y.); shiyaojiang7@gmail.com (Y.S.); 4 Faculty of Liberal Arts, Northwest University, Xi’an 710069, China; zhangjinggujiao@163.com; 5 Rural Education Action Program, Freeman Spogli Institute for International Studies, Stanford University, Stanford, CA 94305, USA; 6 School of Economics and Management, Wuhan University, Wuhan 430072, China; yby110201@126.com; 7 School of Economics, Northwest University of Political Science and Law, Xi’an 710122, China; liuhongyanceee@gmail.com; 8 School of Economics and Finance, Xi’an Jiaotong University, Xi’an 710061, China; 9 Institute of Social Medicine and Health Administration, School of Public Health, Shandong University, Jinan 250012, China; zhouchengchao@sdu.edu.cn; 10 Department of Health Policy and Management, Gillings School of Global Public Health, University of North Carolina at Chapel Hill, Chapel Hill, NC 27599, USA; sean_sylvia@unc.edu

**Keywords:** standardized patients, quality of tuberculosis care, rural-to-urban migrants, migrant clinics

## Abstract

Large and increasing numbers of rural-to-urban migrants provided new challenges for tuberculosis control in large cities in China and increased the need for high quality tuberculosis care delivered by clinics in urban migrant communities. Based on a household survey in migrant communities, we selected and separated clinics into those that mainly serve migrants and those that mainly serve local residents. Using standardized patients, this study provided an objective comparison of the quality of tuberculosis care delivered by both types of clinics and examined factors related to quality care. Only 27% (95% confidence interval (CI) 14–46) of cases were correctly managed in migrant clinics, which is significantly worse than it in local clinics (50%, 95% CI 28–72). Clinicians with a base salary were 41 percentage points more likely to demonstrate better case management. Furthermore, clinicians with upper secondary or higher education level charged 20 RMB lower out of pocket fees than less-educated clinicians. In conclusion, the quality of tuberculosis care accessed by migrants was very poor and policies to improve the quality should be prioritized in current health reforms. Providing a base salary was a possible way to improve quality of care and increasing the education attainment of urban community clinicians might reduce the heavy barrier of medical expenses for migrants

## 1. Introduction

Major improvements have been made in tuberculosis (TB) management in China during recent decades. Prevalence of smear-positive tuberculosis (TB) and TB-related mortality in China have been on the decline, decreasing by 63% and 80%, respectively, from 1990 to 2010 [1]. This is in the wake of several government health reforms, including the scale up of tuberculosis control programs and the introduction of three major health insurance programs that have led to more than 95% of Chinese citizens possessing basic coverage [1,2]. However, despite recent progress in improving health care delivery, China still has the third highest number of TB incident cases in the world [3]. Therefore, TB management in China remains a priority for international and Chinese public health teams. China still has a long way to go to achieve the goal outlined in the new post-2015 End TB Strategy by 2025 [4].

About 247 million migrants have moved from rural to urban areas for higher-wage jobs, and this population of migrants comprises a significant and increasing portion of the total national population in China (18%), which presents new challenges for TB control in large cities [5,6,7]. The rapid influx of migrants, combined with inadequate investment in fundamental public health infrastructure in these communities, has created high incidence rates and low detection and treatment rates for infectious diseases including TB [8,9]. Management of TB in migrant communities presents a unique challenge for several reasons. First, migrants usually share crowded living conditions with poor sanitation [8]. Second, due to limitations of the household registration system (Hukou), many migrants lack medical insurance and pay out of pocket for care, leading migrants facing budget constraints to choose less expensive private and community-level clinics over other sources of care [10]. Migrants also lack knowledge about the disease and have a greater delay between the onset of symptoms and clinical diagnosis [11,12,13,14]. Limited leisure time and absence of sick days aggravate poor healthcare utilization by migrants [15]. These factors contribute to the prevalence of TB in migrant communities increase the need for high quality care for this population. It is essential that clinics in urban migrant communities adequately diagnose and treat tuberculosis to reduce inequities in healthcare utilization [16].

Studies using patient satisfaction surveys have shown migrants to be less satisfied with primary care in comparison to local residents [17]. Other studies using patient perception of health service quality found that migrants gave higher scores for tertiary and secondary hospitals than for primary care clinics located in their communities [18,19]. Although patient satisfaction and perception of quality are important, they do not provide objective measures of quality because satisfaction and perception are subjective and patient recall may be biased. In addition, varying interpretations of quality of care due to patients’ differing medical knowledge, different disease cases and other characteristics make it very hard to compare the quality of care between clinics. However, previous research has rarely taken a relatively objective approach to examining quality of care in urban clinics [20,21,22]. Even less research has focused on health care in urban migrant communities [6,7,23].

This study uses standardized patients (SPs) to avoid the influence of differences in patient disease, patient characteristics and recall bias when comparing quality between different clinicians. SPs were extensively trained to present a specific, consistent case of a certain illness to multiple unsuspecting clinicians in order to measure how clinicians act in organic interactions. SPs have been used to empirically evaluate clinical practice in developed countries since the 1950s [24]. In the past decade, SP methodology has been applied to medical practice in the developing world [25,26,27,28,29,30] and has been previously validated in studies of quality care in China [22,23,31]. SP methodology offers unique advantages compared to other methods of evaluating clinical practice [28,32,33,34]. The first advantage is that observing clinicians who are not aware of being observed produces a more accurate evaluation of their practice [22,34,35]. Another advantage is the ability to compare results across clinicians since the disease is presented in the same way at every visit.

The main goal of this paper is to provide objective evidence on the quality of TB care in urban migrant community clinics. We approach this goal in three ways. First, we describe the characteristics of clinics in migrant communities. We compare differences between clinics that mainly serve migrant workers (here on referred to as “migrant clinics”) and clinics that mainly serve local urban residents (referred to as “local clinics”). Second, we evaluate the quality of TB care provided by both migrant and local clinics using SP visits. To interpret the results of SP visits, we compare the observed practice of clinicians to the International Standards for Tuberculosis Care (ISTC) and Chinese national standards for the diagnosis and treatment of tuberculosis [36,37,38]. Finally, we look at factors that influence clinicians’ practice. In addition to type of clinic, we look at how clinician characteristics such as education, salary, and facility characteristics can lead to high or low quality of health care.

## 2. Materials and Methods

Ethical approval: Approval was granted by the institutional review boards of Stanford University, USA (protocol number: 25904) and Sichuan University, China (protocol number: K2015025). All clinicians who participated in the study gave informed verbal consent during the initial facility survey conducted approximately ten days before SP visits. Clinicians were asked to consent to visits by SPs “at some point in the next month”. Every individual in the role of SP was given instructions on how to avoid invasive tests or procedures.

### 2.1. Selection of Facilities, Data Collection, and Study Size

The results presented here were from a cross-sectional survey on quality of care in urban migrant communities of Shaanxi province in northwestern China. The sample for this study was drawn from one prefecture (the administrative level below the province and above the county) in Shaanxi and the sample prefecture has an overall prevalence of TB (urban and rural combined) on par with the national average [39]. 

The 48 clinics included in this study were selected from two of nine total urban districts in the selected prefecture. Twenty-four migrant communities were chosen out of a list of migrant communities suggested by locals in two selected districts. Since both migrants and locals live together in these communities, we wanted our sample to include both clinics that mainly serve migrant populations (migrant clinics) and clinics that mainly serve local populations (local clinics). To do so, we used a random sampling procedure to identify sample households in each migrant community. After ascertaining the location of the community committee, we used a website to randomly generate another latitude and longitude point within one kilometer of the community committee, and went to the household located nearest to this point to conduct a short household survey. After finishing this survey, our enumerators were instructed to exit the house, turn left, and count three households down the road. This household then became our next sample household. This procedure continued until we had five migrant and five local residents in each community. For example, if the first five households were all migrants, any migrant households after that would be skipped and the procedure would continue until five local resident households were found. To generate a list of candidate clinics, we asked the respondents to name the top three clinics or hospitals they usually visited when they were sick. Based on the clinic list, we contacted clinics with the highest frequency of responses until we found two clinics in each community (48 clinics in 24 communities) that agreed to be visited by SPs at some point in the next month. The communities represented by this sample serve a population of 475,000 migrants [40].

**Figure 1:**
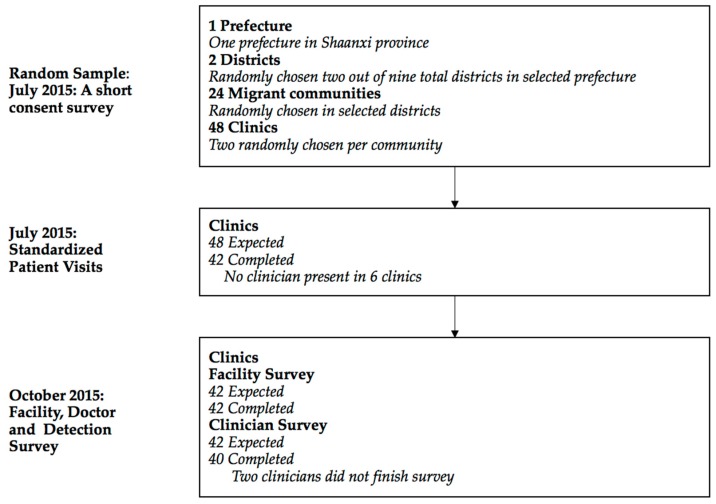
Flow of participants though study.

We conducted three separate waves of data collection (Figure 1). The first wave of data collection consisted of an initial short facility survey that was conducted in early July 2015. Clinician consent for SP visits was obtained at this time. Actual SP visits started ten days after the initial survey in late July 2015. SPs simulating symptoms of TB visited clinicians in all sampled clinics. SPs were randomly assigned to clinics. Within each clinic, SPs visited the clinician following the normal procedures for any walk-in patient. Given a choice of which clinician to visit, SPs randomly chose a clinician following a pre-determined randomization protocol. Our study was therefore designed to represent the care a walk-in patient would receive at each of the sampled clinics. Finally, in October 2015, we conducted a follow-up survey with those clinicians visited by SPs. In this survey, we asked clinicians whether they had detected any SPs and administered a detail facility and clinician survey. This survey collected information including number of patients, number of clinicians, total value of equipment in facility, and clinician’s age, gender, education, medical certification, and salary.

### 2.2. Standardized Patients

The SP case used in this study was adapted to our previous study [22]. In this presumptive TB case, the SP presents with a “cough that is not getting better, and a fever”. Upon appropriate questioning by the clinician, the SP reveals symptoms consistent with a classic case of presumed tuberculosis including a cough duration of 2–3 weeks, fever with night sweats and loss of appetite and weight. The SPs, although recruited specifically to fit the TB case in terms of health and physical characteristics, differed in age, gender, height and weight. Our recruitment standard for TB SPs was that they be around 35 years old, of average weight and height, and healthy with no obvious signs of illness or other conditions that could prejudice diagnoses. The average age of recruited SPs was 37; the youngest was 28 and the oldest 43. Twenty-four SPs (thirteen male, eleven female) were selected after intensively training over a two-week period to consistently present the case to clinicians in the sample.

Following the interaction, SPs purchased all medications prescribed and paid clinicians their usual fee. After each visit, SPs were debriefed using a structured questionnaire and SP responses were confirmed against a recording of the interaction taken using a concealed recording device. Our previous study provides further details and the SP case script (in Mandarin with an English translation) [22]. 

### 2.3. Outcomes

Whether the case was correctly managed and evaluated was assessed in comparison to national standards for TB Care in China and the International Standards for TB Care, 3rd Edition [36,37,38]. The principal outcome of interest was correct case management, which we followed previous researchers Das et al. and Sylvia et al. in defining as the meeting of one of three criteria: referral (either verbal or written) to an upper level health system provider or the Chinese Center for Disease Control and Prevention; recommendation for a chest radiograph; or recommendation for further sputum testing for TB (i.e. smear microscopy, PCR, or culture) [22,26]. The reason that we chose a lenient definition of correct management is that unnecessary and/or harmful medicines are not penalized—a stricter definition would exclude from correct management those cases where additional (unnecessary or even harmful) medicines were given, reducing the proportion of correct management by a significant margin. However, we report the use of (unnecessary) antibiotics, and fluoroquinolones. Antimicrobial resistance is a severe public health concern in China while fluoroquinolones tend to mask underlying TB [41,42,43]. We also collected information on the charged fees of each visit. Since all SPs did not have any medical insurance during visits, all fees charged by clinicians were out of pocket.

In addition to whether SPs were correctly managed, we also assess the clinicians’ adherence to a pre-specified checklist of recommended questions and examinations. We also indicate a short list of “essential” items according to experts from the Center of Tuberculosis Control, Chinese Center for Disease Control and Prevention.

### 2.4. Statistical Analysis

Since we want to compare quality of TB care between migrant clinics and local clinics, an appropriate criterion to distinguish from clinic types is very important. We used data from the household survey in migrant communities. If one clinic was only mentioned by either migrants or local residents in their list of top three frequently visited clinics in the community, the clinic was defined a migrant clinic or a local clinic, respectively. However, some clinics were mentioned by both types of households. For those clinics, we counted the number of times each clinic was mentioned as a households’ first choice. A clinic was defined as a migrant clinic if it was mentioned more times by migrant workers than local residents, and vice versa. If there was a tie for first choice clinics, we counted the second choice, and then the third choice. Using this criteria, all 48 clinics were divided into 30 migrant clinics and 18 local clinics. 

We calculated the proportion or mean and 95% CI by facility type (migrant or local) for correct case management and its individual components (referral, chest radiograph or sputum test) as well as for the use of antibiotics and fluoroquinolones, and the number and percent of checklist items completed. 

To assess the difference in case management between clinic types and SP interactions, we used logistic regressions for binary dependent variables and Ordinary Least Squares (OLS) regressions for continuous dependent variables. We also used logistic and OLS regressions to assess correlations between clinician and facility characteristics and case management outcomes. Two models were used in correlations analysis. The first model included the type of clinic and clinician characteristics: age, gender, education, physician certification and whether the clinician received a base salary. The second model additional included facility characteristics: the number of physicians in the facility, the total value of equipment and whether the building was owned by clinicians. We report these results as margins effects in logistic regressions and coefficients with standard errors in OLS regressions. All analyses were done using Stata 14 (Stata Corporation, College Station, TX, USA).

## 3. Results

Completion rates for each phase of data collection are shown in Figure 1. The short consent survey was completed in all 48 clinics included in the sample. Out of 48 attempted SP interactions, 42 were successfully completed (16 in local clinics and 26 in migrant clinics). No clinicians were present at the time of SP visits six times in sampled clinics. Of the 42 completed SP interactions, matching facility survey was all completed, and clinician survey was completed for 40.

### 3.1. Primary Description of Facilities

The facility survey suggested better facility infrastructure at local clinics in comparison of migrant clinics (Table 1). For instance, although on overage, migrant clinics had 0.34 more full-time staff, there were 0.5 more full-time clinicians in local clinics. Similarly, the total value of equipment was higher in local clinics, at 14,800 RMB, compared to 10,100 RMB in migrant clinics. In addition, 56% clinicians in local clinicians received tuberculosis-specific training in 2014 compared to 33% in migrant communities.

In contrast, migrant clinics received many more patients last year than local clinics: 9153 versus 5858. Salary paid to clinicians in migrant clinics was 7150 RMB per month, compared to only 3060 RMB in local clinics. Furthermore, 88% clinicians in migrant clinics had an upper secondary or higher education attainment, compared to 69% in migrant clinics. There was no large difference in clinicians’ age, physician certification or received base salary.

### 3.2. Case Management of SPs

Table 2 shows the outcomes of interactions between SPs and clinicians in migrant communities. Overall, 15 out of 42 (36%, 95% CI 23–51) cases were correctly managed at clinics in migrant communities. Patients were considered correctly managed if the clinician suggested a chest radiograph (29%, 95% CI 17–44) or a sputum smear test (2%, 95% CI 0–12), or made a referral (29%, 95% CI 17–44). Though not considered incorrect in our lenient definition of correct management, frequent prescriptions of antibiotics unrelated to the treatment of TB were common, occurring in 57% (95% CI 42–71) of interactions, as were 5% (95% CI 1–16) prescriptions for fluoroquinolone. Patients were charged an average of 15.31 (95% CI 8.25–22.1) RMB (2.36 USD). Clinicians spent 9.44 (95% CI 7.23–11.65) minutes on average for the interactions with SPs, and only completed 17% (95% CI 14–20) of checklist items based on ISTC standard and 21% (95% CI 18–25) for China national standard. 

**Figure 2:**
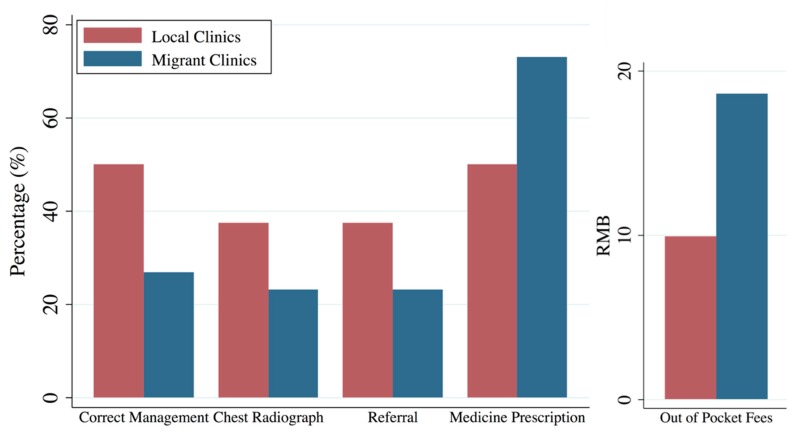
Differences of SP main outcomes between local and migrant clinics.

As shown in Table 2 and Figure 2, quality of TB care provided by local clinics for SPs was significantly better than in migrant clinics. SPs were correctly managed in eight out of 16 interactions (50%, 95% CI 28–74), compared to seven out of 26 interactions in migrant clinics (27%, 95% CI 14–46). This higher correct management rate in local clinics compared to migrant clinics mainly came from suggesting a chest radiograph (38% versus 23%) and referring SPs to higher level hospitals (38% versus 23%). Although unnecessary prescription, because there was no anti-TB medicine available in clinic, were highly frequent in both types of clinics, clinicians in migrant clinics significantly gave more unnecessary prescriptions than those in local clinics: 73% (95% CI 54–86) versus 50% (95% CI 28–72). In addition, the combined out of pocket fees of consultation and medicines was significantly higher in migrant clinics at 18.62 (95% CI 8.82–28.41) RMB (2.86 USD), compared to 9.94 (1.21–18.67) RMB (1.53 USD) in local clinics. Furthermore, clinicians in local clinics asked slightly more questions and performed more examinations during the consultation process according to both ISTC and China national standards than clinicians in migrant clinics.

**Figure 3:**
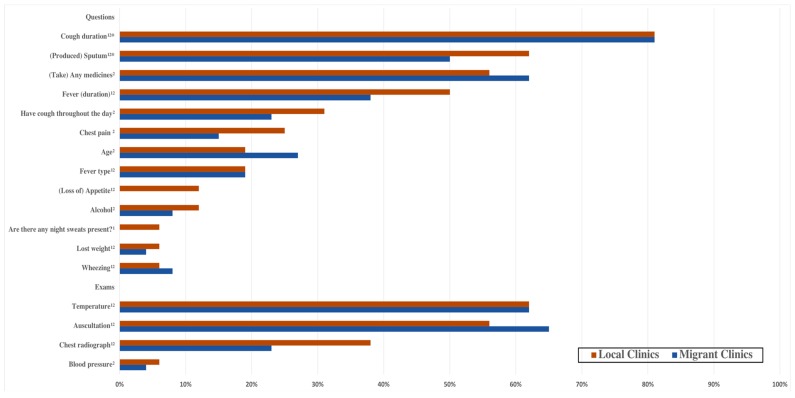
Differences of the Completion of Checklist Items between local and migrant clinics. Notes: ^1^ Chinese Standard; ^2^ International (ISTC) Standard; * essential items.

Completion of each checklist item is shown in Figure 3 and Table A1. More than half of clinicians in both types of clinics performed auscultations, measured temperature, and inquired about cough duration and medications taken. The most common checklist item completed was cough duration, one of four items considered essential questions, asked by 81% of clinicians in both types of clinics. However, two checklist items indicated as essential questions—past tuberculosis and family tuberculosis—were not asked by any clinicians. Fifty percent of clinicians in migrant clinics asked about the production of sputum, another essential question, versus 62% of clinicians at local clinics. Furthermore, questions about fever duration were asked by 38% of clinicians at migrant clinics, which is much less compared to 50% of clinicians in local clinics.

### 3.3. Correlations Between the Quality of Tb Care and Clinic Characteristics

Table 3 shows the factors correlated with quality of TB care. After controlling for clinic types, clinicians with upper secondary or higher education level charged 20 RMB out of pocket fees lower than less-educated clinicians, which is even larger than the difference caused by clinics types, at 13.30 RMB. In addition, clinicians with a base salary were 41% more likely to demonstrate better case management which was mainly contributed by being 48% more likely to refer patients to higher level hospitals, and significantly charged 22 RMB lower out of pocket fees, compared to those clinicians with salary all composed from medical consultation and medicine sales. In addition, in clinics with more clinicians, SPs were more likely to be referred to higher-level hospitals, while SPs were less likely to be referred if the total value of the equipment in the clinic was higher.

## 4. Discussion

Our study used SPs to evaluate the management of TB among healthcare clinicians in rural-to-urban migrant communities in China. The rate of correct case management detected by our study was 36%, indicative of poor quality TB care. Clinicians indicated a preference for radiograph over microbiological testing in contrast to international and national recommendations for TB diagnosis, as radiograph often yield significant false positives [36,37,38]. The poor quality of TB management we found is consistent with results of a previous study evaluating the same disease case in rural China [22]. Poor-quality TB case management contributes significantly to the problem of delayed detection of TB in rural and urban China [11,12,14].

Deficits in TB care were significantly different between migrant and local clinics, consistent with previous literature studying patient perception of care for migrants [18,19]. Comparing to previous literature studying the quality of TB care in rural China, although the rates of correct case management was higher in local clinics than in township health centers which make up the middle tier of China’s rural health system, the rates of correct management in migrant clinics was even lower than rates in rural village clinics, meaning migrants did not share the benefits of higher quality of care in the urban health system, even when they migrated from rural to urban areas [10,22]. The lower quality of TB care delivered by migrant clinics exacerbated gaps in health care equality between migrants and local residents [6,14]. Our results indicated that improving the quality of migrant TB care should be prioritized in current health reforms.

Aside from clinic types, we found clinicians with higher educational attainment significantly charged lower out of pocket fees, mitigating the budget barrier for migrants [14]. This is consistent with previous studies that reiterate ongoing reforms increasing the educational attainment of primary care providers in China [2,35]. We also found that clinicians with a fixed base salary were more likely to refer patients to an upper-level clinician and charged lower fees, indicating they made less profit from patient visits, compared to clinicians without a base salary. In China, clinicians highly depend on profits from medicine sales to supplement their nominal salary [21,44]. Research has shown that physician-induced demand is in part responsible for the high rates of unnecessary medication (e.g., antibiotics) prescriptions in China [42,45]. This suggests a base salary could be a potential way to discourage over-prescription and encourage appropriate referral, a direction in which China seems to be moving [2,20,21]

### Limitations

There are several limitations to our study. First, we evaluated the management of a single SP case depicting a classic presentation of presumptive TB. As a result, we cannot ascertain how clinicians may deal with more complicated TB presentations (e.g., patients with recurrent TB symptoms and suggestive of drug-resistant TB), and whether clinicians can correctly treat known cases of TB. Das et al. (2015) [26], for instance, evaluated clinician management of four different cases including a confirmed case of TB and suspected MDR-TB.

Second, we evaluated one-time new patient interactions. Due to increased risk of detection, SPs did not complete follow-up visits with the 17% of clinicians who asked patients to return (see Table 2). Moreover, SPs were completely unknown to clinicians; clinician treatment of known, regular patients may differ. Differences of stability in patient–clinician relationships were more likely in local clinics than in migrant clinics considering the high mobility of migrant workers [10].

Third, we selected clinics and defined their types from a simple survey of households in migrant communities, however we did not collect more information on patient sorting behavior and health status of both migrants and local residents. A survey of both migrants and local residents in the future would lead to deeper understanding of their choices for clinics.

Finally, although we selected these migrant communities to meet our research design in consideration of the geographical variation of migrants, our study sample was not randomly representative of urban migrant communities.

## 5. Conclusions

Clinics in urban migrant communities provided poor quality care in the diagnosis and management of a classic case of presumptive TB. There was a large gap in correct case management between migrant clinics and local clinics, reinforcing that policies to improve the quality of migrant TB care should be prioritized in current health reforms. Given the significant correlations with correct case management and out of pocket fees, providing a base salary is a possible way to improve quality of TB care and increasing the education attainment of urban community clinicians might help reduce the heavy barrier of medical expenses for migrants.

## Figures and Tables

**Table 1:** Facility and clinician characteristics.

	Full Sample	Local Clinics	Migrant Clinics	Local vs. Migrant *p*-Value
	(N = 42)	(N = 16)	(N = 26)	
**Panel A: Facility Characteristics**				
Number of patients received in 2014	7866.73 (2666.13–13,067.34)	5857.5 (3578.6–8136.4)	9152.64 (527.52–17,777.76)	0.539
Average distance to patients who visit clinic	1.59 (1.18–2.01)	1.43 (0.89–1.96)	1.7 (1.09–2.31)	0.517
Number of staffs working full time at the facility	3.27 (2.47–4.07)	3.06 (2.01–4.12)	3.4 (2.21–4.59)	0.684
Number of clinicians working full time at the facility	2.07 (1.59–2.56)	2.38 (1.44–3.31)	1.88 (1.3–2.46)	0.321
Total value of equipment (10,000 RMB)	1.2 (0.79–1.6)	1.48 (0.65–2.31)	1.01 (0.57–1.46)	0.266
Physicians who own their own facilities	5 (12%, 5–26%)	3 (19%, 7–43%)	2 (8%, 2–25%)	0.318
**Panel B: Clinician Characteristics**				
Age (years)	28 (70%, 55–82%)	10 (63%, 39–82%)	18 (75%, 55–88%)	0.792
Male clinicians	32 (80%, 65–90%)	11 (69%, 44–86%)	21 (88%, 69–96%)	0.401
Clinicians with upper secondary or higher education	21 (53%, 37–67%)	8 (50%, 28–72%)	13 (54%, 35–72%)	0.158
Clinicians with Practicing Physician Certificate	5 (13%, 5–26%)	2 (13%, 3–36%)	3 (13%, 4–31%)	0.796
Clinicians with Assistant Practicing Physician Certificate	11 (28%, 16–43%)	6 (38%, 18–61%)	5 (21%, 9–40%)	––
Clinicians with Rural Physician Certificate	28 (70%, 55–82%)	11 (69%, 44–86%)	17 (71%, 51–85%)	0.253
Clinicians receiving base salary	28 (70%, 55–82%)	10 (63%, 39–82%)	18 (75%, 55–88%)	0.888
Monthly salary (1000 RMB)	5.51 (1.25–9.76)	3.06 (1.55–4.56)	7.15 (–0.02–14.31)	0.347
Clinicians who received Tuberculosis-specific training in 2014	17 (43%, 29–58%)	9 (56%, 33–77%)	8 (33%, 18–53%)	0.155

Notes: Data are mean (95% CI) for continuous variables and number (mean, 95% CI) for dummy variables.

**Table 2:** Main outcomes of interactions with standardized patients.

	Full Sample	Local Clinics	Migrant Clinics	Local vs. Migrant *p*-Value
**Patient-clinician interactions**	42	16	26	
**Case management**				
Correctly managed the case ^§^	36	50	27	0.086
	(23–51)	(28–72)	(14–46)	
Ordered a chest radiograph	29	38	23	0.290
	(17–44)	(18–61)	(11–42)	
Ordered a sputum smear test	2	0	4	0.497
	(0–12)	(0–19)	(1–19)	
Referred to higher level hospitals	29	38	23	0.179
	(17–44)	(18–61)	(11–42)	
Referred to CDC or DOTs, if referral	2	0	4	0.497
	(0–12)	(0–19)	(1–19)	
Medicine Prescribed	64	50	73	0.024
	(49–77)	(28–72)	(54–86)	
Asked patient to return	17	6	23	0.141
	(8–31)	(1–28)	(11–42)	
Gave any antibiotic	57	50	62	0.282
	(42–71)	(28–72)	(43–78)	
Gave any fluoroquinolone	5	6	4	0.907
	(1–16)	(1–28)	(1–19)	
**Process**				
Time with clinician (min)	9.44	9.16	9.61	0.449
	(7.23–11.65)	(5.47–12.85)	(6.66–12.57)	
Number of questions and examinations (ISTC)	5.38	5.5	5.31	0.828
	(4.48–6.28)	(3.75–7.25)	(4.22–6.4)	
% of questions and examinations (ISTC)	17	18	17	0.828
	(14–20)	(12–23)	(14–21)	
Number of questions and examinations (China)	3.81	4	3.69	0.663
	(3.18–4.44)	(2.77–5.23)	(2.95–4.44)	
% of questions and examinations (China)	21	22	21	0.663
	(18–25)	(15–29)	(16–25)	
% of essential history checklist asked by clinician (Both Standards)	34	36	33	0.739
	(29–39)	(26–46)	(26–40)	
Out of pocket fees of consultation and medicines combined (RMB)	15.31	9.94	18.62	0.024
	(8.52–22.1)	(1.21–18.67)	(8.82–28.41)	
Out of pocket fees of consultation and medicines combined (US dollars) *	2.36	1.53	2.86	0.024
	(1.31–3.4)	(0.19–2.87)	(1.36–4.37)	
**Diagnosis**				
Mentioned tuberculosis	5	6	4	0.182
	(1–16)	(1–28)	(1–19)	

Notes: Data are mean or percent (95% CI). * 1 USD = 6.5 RMB. **^§^** Correctly managed for Tuberculosis is defined as a chest radiograph, sputum test, or referral. “ISTC” is ISTC standard and “China” is China national standard.

**Table 3:** Correlates of correct case management, chest radiograph, referral, medicine prescription and out of pocket fees of interactions with standardized patients among local and migrant clinics.

	Correct Case Management		Chest Radiograph		Referral		Medicine Prescription		Out of Pocket Fees	
	(1)	(2)	(3)	(4)	(5)	(6)	(7)	(8)	(9)	(10)
Service for migrant workers	−0.29 **	−0.29 *	−0.28 *	−0.36 **	−0.24 *	−0.30 *	0.29 **	0.32 **	15.52 *	13.30 *
	(0.11)	(0.12)	(0.13)	(0.14)	(0.11)	(0.12)	(0.10)	(0.12)	(6.14)	(6.20)
Clinician age (years)	−0.01	−0.01	−0.01	0.00	−0.00	0.00	0.01	0.01	0.34	0.38
	(0.01)	(0.01)	(0.01)	(0.01)	(0.01)	(0.01)	(0.01)	(0.01)	(0.28)	(0.27)
Male clinician	0.06	0.04	0.03	0.06	0.19	0.17	−0.06	−0.07	−4.27	−8.42
	(0.16)	(0.15)	(0.17)	(0.17)	(0.16)	(0.14)	(0.17)	(0.16)	(7.19)	(7.29)
Clinician education, upper secondary or higher	0.12	0.04	0.08	0.29	0.19	0.20	−0.23	−0.24	−23.48 *	−20.22 *
	(0.22)	(0.24)	(0.27)	(0.34)	(0.22)	(0.23)	(0.23)	(0.26)	(8.62)	(8.59)
Practicing physician certificate	−0.24 *	−0.22	−0.35 **	−0.41 **	−0.21	−0.21	0.13	0.15	4.73	10.81
	(0.12)	(0.12)	(0.12)	(0.15)	(0.13)	(0.12)	(0.14)	(0.14)	(6.17)	(6.33)
Clinician has base salary	0.28	0.41 *	--	--	0.21	0.48 **	−0.01	−0.18	−9.13	−22.00 *
	(0.15)	(0.21)	--	--	(0.15)	(0.18)	(0.16)	(0.22)	(6.95)	(8.39)
Number of clinicians in a clinic		0.06		−0.14		0.12 *		−0.07		−6.52 *
		(0.06)		(0.09)		(0.05)		(0.07)		(2.63)
Total value of equipment (10,000 RMB)		−0.05		0.06		−0.12 *		0.07		0.00
		(0.06)		(0.06)		(0.05)		(0.06)		(2.38)
Building of clinic owned by clinicians		0.15		−0.34		0.04		−0.05		12.99
		(0.23)		(0.25)		(0.18)		(0.23)		(10.19)
Number of Observations	40	40	28	28	40	40	40	40	40	40
Mean of Outcome	0.36		0.29		0.29		0.64		15.31	

Notes: Results are reported as marginal effects of logistic regressions in Columns (1)–(8) and OLS regressions in Columns (9) and (10). Standardized errors are reported in parentheses under coefficients. Correct case management is defined as a chest radiograph or sputum test or referral. * *p* < 0.05, ** *p* < 0.01, *** *p* < 0.001.

## Appendix A

**Table A1:** Completion of Checklist Items.

	Total	Local Clinics	Migrant Clinics
**Questions**			
Cough duration ^1,2,^*	0.81 (0.40)	0.81 (0.40)	0.81 (0.40)
(Produced) Sputum ^1,2,^*	0.55 (0.50)	0.62 (0.50)	0.50 (0.51)
Past tuberculosis ^2,^*	0.00 (0.00)	0.00 (0.00)	0.00 (0.00)
Family tuberculosis ^2,^*	0.00 (0.00)	0.00 (0.00)	0.00 (0.00)
Blood in sputum ^1,2^	0.02 (0.15)	0.00 (0.00)	0.04 (0.20)
Fever (duration) ^1,2^	0.43 (0.50)	0.50 (0.52)	0.38 (0.50)
Fever type ^1,2^	0.19 (0.40)	0.19 (0.40)	0.19 (0.40)
Chest pain ^2^	0.19 (0.40)	0.25 (0.45)	0.15 (0.37)
(Loss of) Appetite ^1,2^	0.05 (0.22)	0.12 (0.34)	0.00 (0.00)
Lost weight ^1,2^	0.05 (0.22)	0.06 (0.25)	0.04 (0.20)
Breathing difficulty ^1,2^	0.05 (0.22)	0.00 (0.00)	0.08 (0.27)
Wheezing ^1,2^	0.07 (0.26)	0.06 (0.25)	0.08 (0.27)
(Take) Any medicines ^2^	0.60 (0.50)	0.56 (0.51)	0.62 (0.50)
Smoking ^2^	0.10 (0.30)	0.00 (0.00)	0.15 (0.37)
Diabetes ^2^	0.00 (0.00)	0.00 (0.00)	0.00 (0.00)
High blood pressure or hypertension ^2^	0.00 (0.00)	0.00 (0.00)	0.00 (0.00)
HIV/AIDS ^2^	0.00 (0.00)	0.00 (0.00)	0.00 (0.00)
Alcohol ^2^	0.10 (0.30)	0.12 (0.34)	0.08 (0.27)
Age ^2^	0.24 (0.43)	0.19 (0.40)	0.27 (0.45)
Family symptoms ^2^	0.00 (0.00)	0.00 (0.00)	0.00 (0.00)
Have cough throughout the day ^2^	0.26 (0.45)	0.31 (0.48)	0.23 (0.43)
Weakness ^1^	0.02 (0.15)	0.00 (0.00)	0.04 (0.20)
Are there any night sweats present? ^1^	0.02 (0.15)	0.06 (0.25)	0.00 (0.00)
**Exams**			
Weight ^1,2^	0.00 (0.00)	0.00 (0.00)	0.00 (0.00)
Pulse ^2^	0.10 (0.30)	0.06 (0.25)	0.12 (0.33)
Blood pressure ^2^	0.05 (0.22)	0.06 (0.25)	0.04 (0.20)
Temperature ^1,2^	0.62 (0.49)	0.62 (0.50)	0.62 (0.50)
Auscultation ^1,2^	0.62 (0.49)	0.56 (0.51)	0.65 (0.49)
Chest radiograph ^1,2^	0.29 (0.46)	0.38 (0.50)	0.23 (0.43)
Sputum smear test (Sputum AFB) ^1,2^	0.02 (0.15)	0.00 (0.00)	0.04 (0.20)
HIV test ^2^	0.00 (0.00)	0.00 (0.00)	0.00 (0.00)
Diabetes test ^2^	0.00 (0.00)	0.00 (0.00)	0.00 (0.00)
Mantoux Tuberculin Skin Test (TST) ^1,2^	0.00 (0.00)	0.00 (0.00)	0.00 (0.00)
Sputum culture test ^1^	0.00 (0.00)	0.00 (0.00)	0.00 (0.00)

Notes: Data are proportion (standard deviation). ^1^ Chinese Standard, ^2^ International (ISTC) Standard, * essential items.
